# Injectable Affinity and Remote Magnetothermal Effects of Bi‐Based Alloy for Long‐Term Bone Defect Repair and Analgesia

**DOI:** 10.1002/advs.202100719

**Published:** 2021-05-20

**Authors:** Yuanyuan He, Yu Zhao, Linlin Fan, Xuelin Wang, Minghui Duan, Hongzhang Wang, Xiyu Zhu, Jing Liu

**Affiliations:** ^1^ Department of Biomedical Engineering School of Medicine Tsinghua University Beijing 100084 China; ^2^ Orthopedic Department Second Hospital of Shanxi Medical University Taiyuan Shanxi 030001 China; ^3^ Technical Institute of Physics and Chemistry Chinese Academy of Sciences Beijing 100190 China; ^4^ School of Engineering Medicine Beihang University Beijing 100191 China; ^5^ Interdisciplinary Institute for Cancer Diagnosis and Treatment Beijing Advanced Innovation Center for Biomedical Engineering Beihang University Beijing 100191 China

**Keywords:** bone defect repair, hyperthermia analgesia, injectable Bi alloy, metallic graft materials, non‐invasive wireless energy delivery

## Abstract

As alternatives, metallic/nonmetallic bone graft materials play significant roles in bone defect surgery to treat external trauma or bone disease. However, to date, there are rather limited long‐term implantable materials owning to in situ molding incapability of metallics and poor mechanical property of nonmetallics. Here, Bi‐based low melting point alloy, with unique properties of injectability, solid‐liquid phase transition, mechanical capability, and biocompatibility, present obvious long‐lasting bone affinity as the excellent artificial bone‐substitute. It is particularly necessary to point out that the targeted injected Bi alloy remains in its original position for up to 210 days without moving, as well as, displays good osseointegration ability to resolve repeated revision trauma caused by losing bone repair material. Additionally, with outstanding electrical and thermal conductivity, an unconventional way using Bi alloy to realize very beneficial hyperthermia analgesia via non‐invasive wireless energy delivery is first proposed, which avoids adverse effects on bone remodeling inflicted by traditional drugs. The significantly decreased expression of pain sensitizing factor, such as, interleukin‐6, neuropeptide substance, and transient receptor potential vanilloid 1 reveals the potential mechanism of hyperthermia analgesia. The present findings suggest the combination therapy of Bi alloy in bone repair and analgesia, which owns far‐reaching clinical application value.

## Graphical abstract

The injectable Bi‐based alloy with good bone affinity enables in repairing bone defects in situ for a long time. At the same time, it can suppress the expression of pain sensitization substance (IL‐6, SP, TRPV1) by remote magnetic heat, so as to realize non‐invasive treatment of acute and chronic pain induced by bone defects.

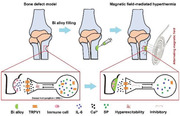

## 1 Introduction

Bones play pretty vital roles in the human body, such as, providing the framework for the attachment of muscles and tissues and protecting internal organs from injury.^[^ ^1^ ^]^ External trauma, bone tumors, and other bone diseases lead to bone defects and induce acute or chronic pain, which are major challenges for bone surgery.^[^ ^2^, ^3^, ^4^, ^5^ ^]^ The bones of those elders who have high incidence of bone diseases or large bone defects are often less capable of regeneration. Such patients need bone grafts to replace the missing bone. It is estimated that ≈2.5 million bone grafts are carried out worldwide each year, and market sales of bone grafts and bone substitutes exceed $2.5 billion.^[^ ^6^ ^]^ Autogenous bone graft has long been regarded as the gold standard of bone repair. However, its desirable range is limited, and there may induce some complications such as chronic pain.^[^ ^7^ ^]^ Moreover, allogeneic bone grafts could be adopted to repair bone defects. However, potential risks exist in immune rejection and pathogen transmission, and the number of bone donors is often less than clinic needs.^[^ ^8^ ^]^ Recently, a variety of bone graft materials such as metallic and nonmetallic materials have been developed to replace the missing bones. Generally, an ideal bone graft material should have similar mechanical properties as the bone at the implantation site and provide a good microenvironment for the adherent growth of new bones.^[^ ^9^, ^10^ ^]^ Traditional metallic bone graft materials such as, gold, silver, cobalt‐chromium alloy^[^ ^11^ ^]^ have been used as bone components. For example, they could be adopted in hip and knee joints to replace necrotic bones for support and weight‐bearing functions. However, these materials have higher elastic modulus than bone tissue. And they have to be replaced after a certain period of time, due to causing aseptic loosening at interfaces, which would make patients feel pain.^[^ ^6^, ^11^ ^]^ As for the high melting point, they have expensive production cost and poor shape plasticity due to being unable to be injected into bone defects and have to be made into specific shapes before use.

**Scheme 1:**
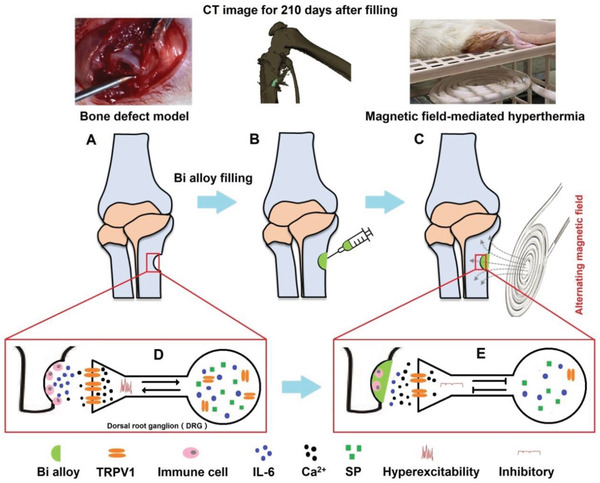
Schematic illustration of Bi alloy‐based bone defect filling and analgesia. A) Bone defect model construction. B) Treatment of bone defect by filling Bi alloy with a heated syringe. Micro‐CT image shows metal filling position (the fillers in bone marked green represent Bi alloy). C) The temperature of Bi alloy was controlled remotely with AMF to suppress pain. D) Bone defect leads to increased expression of pain sensitizing factor (IL‐6, SP) and pain receptor (TRPV1), and enhanced pain signal transmission. E) After magnetic hyperthermia, sensitization factors and pain signals are inhibited and pain is released.

As an alternative, the recently emerging low‐melting‐point metals display excellent properties such as low melting point, plastic shape, good biocompatibility, as well as, the basic characteristics of traditional metals including strong mechanical properties, good electrical, and thermal conductivity. Such metallic materials are becoming a hot focus in the fields of 3D printing, flexible electronics, and translational biomedical sciences.^[^ ^12^ ^]^ This feature of low melting enables the metal to achieve multimodal transformation with low energy and cost consumption. That is, they are more likely to do plastic deformation when subject to temperature changes. For example, gallium based liquid metal behaves just like water, kaleidoscopic.^[^ ^13^ ^]^ The bismuth‐based alloy with a melting point of about 60 °C can be used as a 3D printing material to produce a variety of 3D graphics,^[^ ^14^ ^]^ or as a potential bone cement for bone injuries.^[^ ^15^ ^]^ Bone cements have been introduced in the clinical setting to fill bone defects. However, traditional bone cements would generate a relatively high temperature during the solidification process, which might cause certain damage to the surrounding bone tissue, and the risk of cement leakage.^[^ ^16^, ^17^, ^18^ ^]^ Low melting point metals in solid state show strong mechanical properties to replace gypsum for shaping or making mechanical arms.^[^ ^19^ ^]^ In this work, Bi alloy (Bi_35_In_48.6_Sn_16_Zn_0.4_) as a low melting point metal was introduced as a bone repair material candidate for bone defects because of its diverse merits (**Scheme** **1**A,B). The implantation position and movement of Bi alloy after surging and injecting were observed by Micro‐CT imaging. It was surprisingly found that the position of Bi alloy in bone filling site observed on the 210th day after bone grafting did not move significantly (Scheme 1B). Moreover, the bone‐metal section was applied to show the long‐term affinity on the bone‐metal surface.

In addition, with increasingly being paid attention to thermotherapy for analgesia, hot dry needles or electric heating needles have been applied to treat chronic shoulder pain or prostate inflammation by means of current heat transfer,^[^ ^20^, ^21^ ^]^ especially pulsed radiofrequency treatment of chronic pain such as occipital neuralgia, radicular pain, trigeminal neuralgia, knee, and shoulder pain showing superior outputs over conventional radiofrequency or steroid injections.^[^ ^22^ ^]^ However, this kind of treatment will lead to tissue trauma and its treatment depth is limited. Alternation magnetic field (AMF)‐triggered treatment of tumors through heat conduction can realize noninvasive operation and remote control.^[^ ^23^ ^]^ Low melting point metals were demonstrated to be a powerful magnetothermal agent for AMF because of their high electrical and thermal conductivity. Herein, Bi alloy was utilized to alleviate mechanical and thermal pain sensitivity by remote temperature control based on the strong magnetic thermal effect (Scheme 1C). To clarify the mechanism of hyperthermia analgesia, a series of conceptual experiments were performed. The dorsal root ganglion (DRG) contains the cell bodies of the primary sensory neurons responsible for transducing and transmitting to the sensory center of the brain. It plays a crucial role in traumatic pain, where multiple immune factors and membrane protein receptors involved in pain allergy are highly aggregated, such as, tumor necrosis factor, interleukin‐1*β* (IL‐1*β*), interleukin‐6 (IL‐6), inflammatory mediator (neuropeptide calcitonin gene related peptide), neuropeptide substance P (SP), and pain receptor (transient receptor potential vanilloid 1, TRPV1).^[^ ^4^, ^24^, ^25^, ^26^, ^27^, ^28^, ^29^, ^30^, ^31^ ^]^ In this study, after 5 days of bone injury hyperthermia, DRG sections were taken for immunohistochemical staining. It revealed that magnetic hyperthermia could relieve pain by inhibiting the expression of IL‐6, SP, and TRPV1 (Scheme 1D,E). Furthermore, we conducted a comprehensive biosafety evaluation of the Bi alloy. It was found that no abnormalities occur from cytotoxicity in vitro to safety assessment in vivo for up to 7 months, which gives pretty strong evidence to support future possible applications. To sum up, this Bi alloy implantation combined with AMF therapy could significantly improve bone remodeling and even relieve chronic bone pain in rats.

## 2 Results

### 2.1 Fabrication and Characterization of Bi Alloy

**Figure 1:**
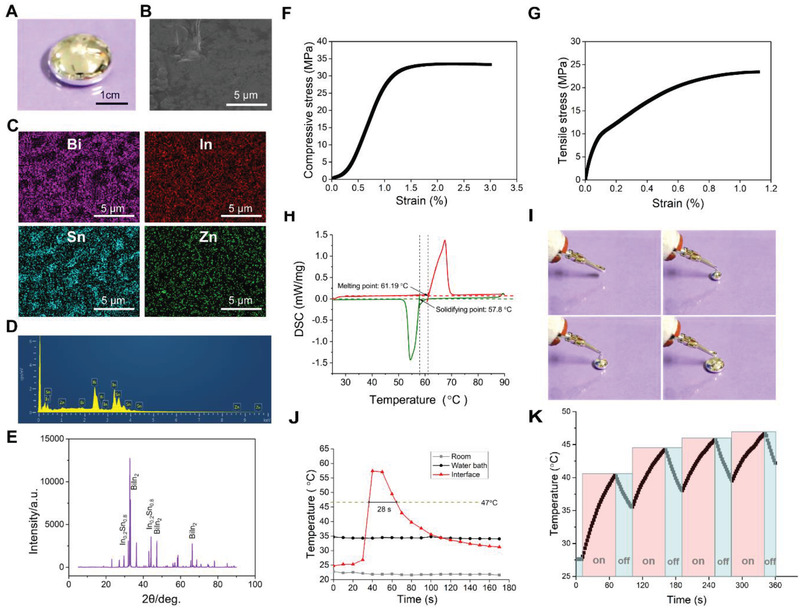
Characterization of Bi alloy (Bi_35_In_48.6_Sn_16_Zn_0.4_). A) The optical photo and B) SEM image of Bi alloy. C,D) are the EDS maps corresponding to (B). E) XRD patterns of Bi alloy. F) Stress–strain curves of Bi alloy under compression. G) Stress–strain curves of Bi alloy in tension. H) DSC curves of Bi alloy. I) Injectability of Bi alloy via heatable syringe. J) The temperature–time curve of Bi alloy injecting process. K) Thermal cycling by switching the AMF ON for 60 s and AMF OFF for 30 s (the test lasted for 6 min).

The Bi_35_In_48.6_Sn_16_Zn_0.4_ (Bi alloy) material was prepared by placing bismuth (Bi), indium (In), tin (Sn), and Zinc (Zn) metals in a vacuum high‐temperature melting furnace with the mass ratio of 35%, 48.6%, 16%, and 0.4%. The surface of Bi alloy has obvious metallic luster (**Figure** **1**A). The microstructure of Bi alloy and the distribution of various metallic elements were observed by scanning electron microscopy (SEM, Figure 1B) and energy‐dispersive spectrometer (EDS, Figure 1C,D). As shown in Figure 1B, the morphology of the Bi alloy is uniform and compact. And the element distribution mapped by EDS confirmed the composition of Bi alloy, showing the existence of Bi, In, Sn, and Zn. The X‐ray diffraction (XRD) patterns of Bi alloy show the prominent formation of BiIn_2_ and In_0.2_Sn_0.8_ phases in the crystalline phase (Figure 1E).

To explore the stability of Bi alloy in liquid environment, the concentration of metallic elements released from Bi alloy after soaking in Hank's solution (HBSS) for 12, 24, and 72 h was tested using a plasma mass spectrometer (IPC‐MS, X Series, Thermo, America). Similar to previous studies, the release of Bi, In, Sn metallic ions was low and stable, but Zn was relatively high and increased with the prolonging of soaking time. However, for the lowest content (0.4%) within alloy, the whole content of Zn in solutions was less (Figure S1, Supporting Information).^[^ ^15^ ^]^ Furthermore, the slow release of Zn helps to promote bone growth,^[^ ^15^, ^32^ ^]^ suggesting that small amounts of dissolved zinc are very safe for living organisms. On the whole, Bi alloy has some corrosion resistance. In addition, after soaking 1, 2, 3, 4, 5, and 6 days, the mass of Bi alloy has no significant change (Figure S1B, Supporting Information), which further indicates that Bi alloy has good stability in liquid environment.

The compressive strength (Figure 1F), tensile strength (Figure 1G), and elastic modulus of Bi alloy were 33.82 ± 0.76, 24.48 ± 0.68, and 115.87 ± 4.39 MPa, respectively, which were matched with cancellous to avoid stress loosening.^[^ ^15^, ^33^ ^]^

The melting point of Bi alloy measured by Differential scanning calorimetry (DSC) was 61.19 °C (Figure 1H), which was consistent with previous literature.^[^ ^14^ ^]^ Due to the low melting point of Bi alloy, it could be injected smoothly through a heated syringe (Figure 1I, Figure S2, Supporting Information). It takes only 28 s for Bi alloy to cool from 61 to 47 °C after melting (as shown in Figure 1J). This melting point temperature did not reach the thermal coagulation limit of collagen (70–72 °C),^[^ ^34^ ^]^ and the duration of high temperature was lower than the thermal necrosis threshold of epithelial cells (52–55 °C, 30 s) and the critical value of bone recovery (47 °C, 60 s).^[^ ^35^ ^]^ Therefore, it was insufficient to cause osteonecrosis and stimulate inflammatory reaction, indicating that the melting heat injection temperature of bismuth alloy is relatively safe.

In other words, the Bi alloy with good physical properties has the potential to be a bone repair material for in situ injection. In addition, due to its excellent electrical and thermal conductivity properties, Bi alloy exhibits excellent magneto‐thermal effects by AMF shown in Figure 1K.

### 2.2 Bi Alloy Implantation for Bone Defect and Micro‐CT Image

**Figure 2:**
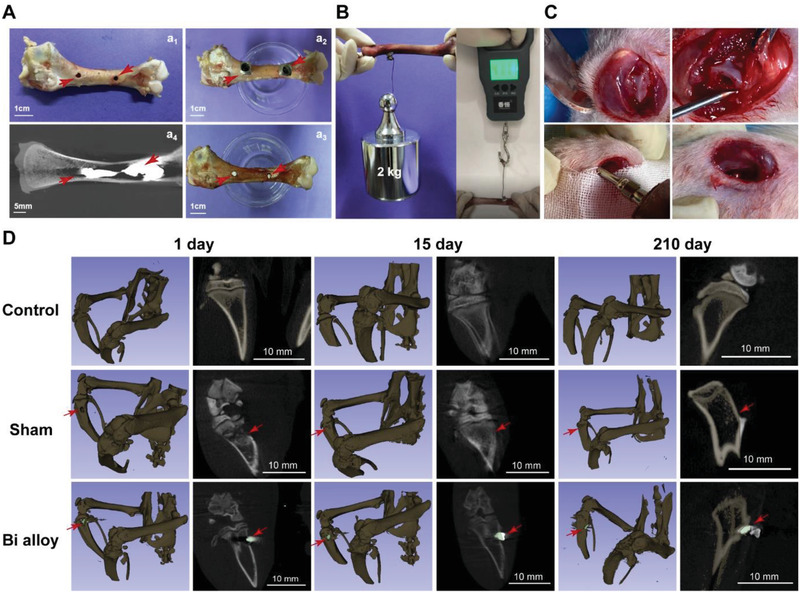
In vitro and in vivo repair of bone defects. A) Bone defect model of chicken legs (a_1_ and a_2_) and the implant of Bi alloy into the bone defect (a_3_), as well as, the Micro‐CT imaging of bone repair (a_4_). B) The binding force of Bi alloy embedded in bone tissue was tested through hanging weights and dynamometer. C) Bone defect model of rat legs and the implant of Bi alloy into the bone defect. D) Micro‐CT images of rat legs about non‐surgical group (control), bone defect group (sham), and Bi alloy implantation into bone defect group (Bi alloy) for 1, 15, 210 days. Arrows show the site of bone defect with Bi alloy or not.

This Bi alloy has low melting point and could undergo liquid–solid phase transformation quickly, which is beneficial to the treatment of bone defects with in situ injection. As shown in **Figure** **2**A, Liquid Bi alloy was injected into the defect sites constructed on the side of the chicken bone in vitro, which filled all of them. Micro‐CT images showed that liquid Bi alloy was injected deep into the bone marrow cavity along the needle, showing good fluidity and filling effect. In addition, the mechanical test showed that the Bi alloy filled in the bone defect could bear more than 2 kg of weight and even resist more than 40 N of force (Figure 2B), which indicated that it had a good binding force with bone. To evaluate its therapeutic effect on bone defects, Bi alloy was injected at the lower knee joint of rat's left leg (Figure 2C). Moreover, the implantation and long‐term displacement of Bi alloy in bone defect could be observed through Micro‐CT imaging due to the radiopacity of Bi alloy. This is conductive to its accurate observation and position. As shown in Figure 2D, the Bi alloy was located on the bone defect site on the first day of implantation, and the Bi alloy did not shift or fall off on the 15th or 210th day. It indicated that Bi alloy can be used for long‐term repair of bone defects.

### 2.3 Affinity of Bi Alloy and Bone

**Figure 3:**
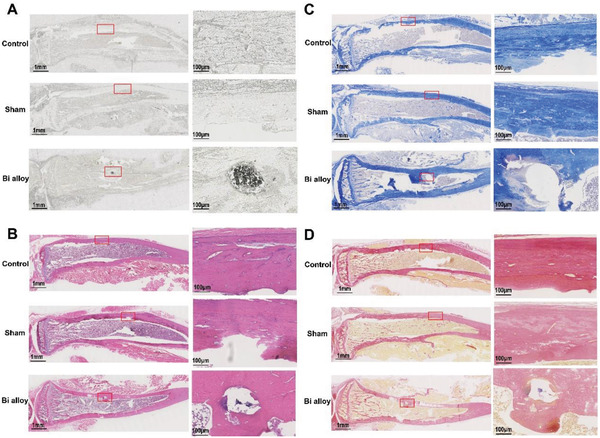
Biocompatibility assessment of no material (control and sham) or Bi alloy in bone defect at 210 days after implantation. A) Unstained tissue sections show that the Bi alloy is intact and maintains a good filling effect at the defect. B) HE staining indicated a more complete structure around the filler Bi alloy and fewer inflammatory cells can be seen around it. C) TB staining shows that dark blue new regeneration bone tissue and pink new osteoblasts can be seen around the filling Bi alloy. D) VG staining shows pink new collagen fibers. All images were taken at 1× and 100× magnification.

To further evaluate whether Bi alloy can be used as a long‐term bone graft material, the affinity of Bi alloy and bone was investigated by hematoxylin‐eosin (HE), toluidine blue (TB), and Van Gieson (VG) staining at the bone defect site after implantation for 210 days. Micro‐CT images showed that the Bi alloy did not fall off after implantation for 210 days (Figure 2C), and bone tissue sections showed that the Bi alloy was closely bound to bone tissue (**Figure** **3**A). This further proved its ability of integration with bones, which was conductive to reduce the repeated revisions. Moreover, HE staining showed that tissues around Bi alloy were intact and a few inflammatory cells were aggregated (Figure 3B). The TB and VG staining showed that there was no significant effect on the surrounding tissue formation and fibrin distribution (Figure 3C,D). Therefore, the Bi alloy could serve as an excellent bone graft material for bone defect repair with low trauma because of its excellent biosafety and affinity.

### 2.4 Hyperalgesia Relief with Alternation Magnetic Field

**Figure 4:**
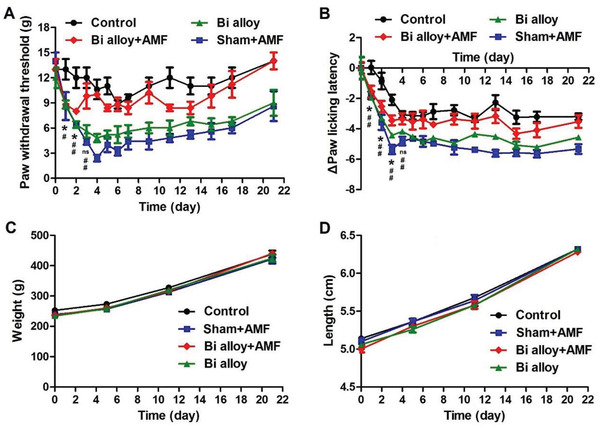
Evaluation of the effects of magneto‐thermal analgesia and thermal stimulation on the physical condition of rats. A) The time course of changes in PWT values after operation was investigated (*n* = 5, ns *p* > 0.05, *p < 0.05; ^#^ *p* < 0.05, ^##^ *p* < 0.01). B) The time course of changes in PLL values after operation was investigated (*n* = 5, ns *p* > 0.05, *p < 0.05; ^##^ *p* < 0.01). C) The change of weight (*n* = 5, *p* > 0.05) and D) leg length (*n* = 5, *p* > 0.05) over time. The obtained data are expressed as mean ± SEM, *p*‐values are calculated using two‐tailed unpaired t‐test.

The bone defect area of rats implanted with Bi alloy was placed directly above the magnetic coil. To treat the pain, the temperature of hyperthermia was controlled by adjusting the power of AMF and the frequency of hyperthermia. The effect of magneto‐thermal therapy on acute and chronic pain induced by bone defect was tested by using Von Frey cilium and hot plate pain measuring instrument. As shown in **Figure** **4**A, compared with the control group, the paw withdrawal threshold (PWT) values in sham+AMF group and Bi alloy groups decreased significantly after bone defect surgery for 1 to 21 days (*n* = 5, ^#^ *p* < 0.05, ^##^ *p* < 0.01). The PWT values in Bi alloy+AMF group decreased significantly on the 1st and 2nd day after surgery. Then, they rose and leveled off compared with the control group from the 3rd day (*n* = 5, ns *p* > 0.05, *p < 0.05). The Bi alloy+AMF group showed significant suppression of PWT values decreasing compared to sham+AMF group and Bi alloy group. Similarly, the paw licking latency (PLL) values in sham+AMF group and Bi alloy groups (*n* = 5, ^##^ *p* < 0.01) decreased significantly after bone defect surgery for 1 to 21 days (Figure 4B). And the PLL values in Bi alloy+AMF group decreased significantly on the 1st, 2nd, and 3rd day after surgery, and backed to normal levels from the 4th day (*n* = 5, ns *p* > 0.05, *p < 0.05). This proved that thermal stimulation does help to relieve pain after bone defect. In addition, the growth status of rats in weight (Figure 4C) and leg length (Figure 4D) was not affected by magneto‐thermal stimulation.

### 2.5 Inhibition of the Expression of Interleukin‐6, Neuropeptide Substance P, and Transient Receptor Potential Vanilloid 1 in Dorsal Root Ganglion Induced by Bone Defect through Magnetic Hyperthermia

**Figure 5:**
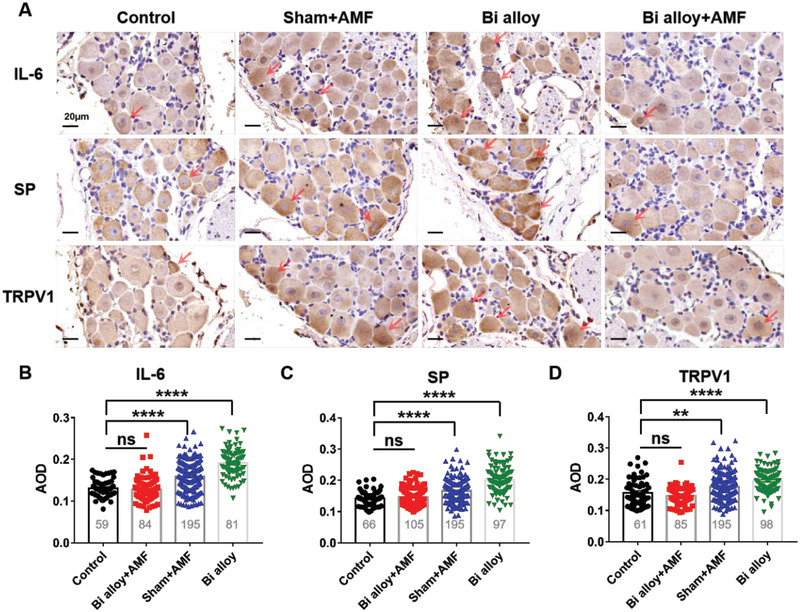
Immunohistochemistry for expression of IL‐6, SP, and TRPV1 in L4‐5 DRG after the treatment of pain by AMF or not among all groups. A) Light micrographs (×40) showing immunohistochemistry for IL‐6, SP, and TRPV1 in L4‐5 DRG. Arrows show some high expressions of them in neurons. Scale bar: 20 µm. Quantification of expression levels of IL‐6, SP, and TRPV1, as shown in (B, C and D), by the average optical density (AOD) of neurons in the staining sections. Results represent mean ± SEM in each group, *n* shown in histogram, *p*‐values are calculated using two‐tailed unpaired t‐test, ns *p* > 0.05, **p < 0.01, ****p < 0.0001, compared to naive control.

To further verify the inhibitory effect of heat regulation on pain, we applied immunohistochemical method to detect the expression of IL‐6, SP, and TRPV1 in DRG. The DRGs (L4‐5) of rats after 5 days of magnetic hyperthermia or not were taken for immunohistochemical staining. Negative results of phosphate buffered saline (PBS) showed uniform background staining of cells within or between groups, indicating no obvious non‐specific staining (Figure S3, Supporting Information). The stain of IL‐6, SP, and TRPV1 in the Sham+AMF and Bi alloy groups were significantly deeper than control group, and the Bi alloy+AMF group was similar to that in the control group (**Figure** **5**A). Statistical analysis showed that the average optical density (AOD) of IL‐6, SP, and TRPV1 in Sham+AMF and Bi alloy groups were significantly higher than control group. However, no significant differences were found between the Bi alloy+AMF and control group (Figure 5B–D). The percentage of positive cells in Sham+AMF and Bi alloy groups revealed increased IL‐6, SP, and TRPV1‐marked neurons compared with control and treatment groups (Figure S4, Supporting Information). These results indicate that the hyperthermia of Bi alloy with AMF significantly inhibits the expression of pain sensitizers, thus alleviating pain symptoms.

### 2.6 Biocompatibility of Bi Alloy In Vitro and In Vivo

**Figure 6:**
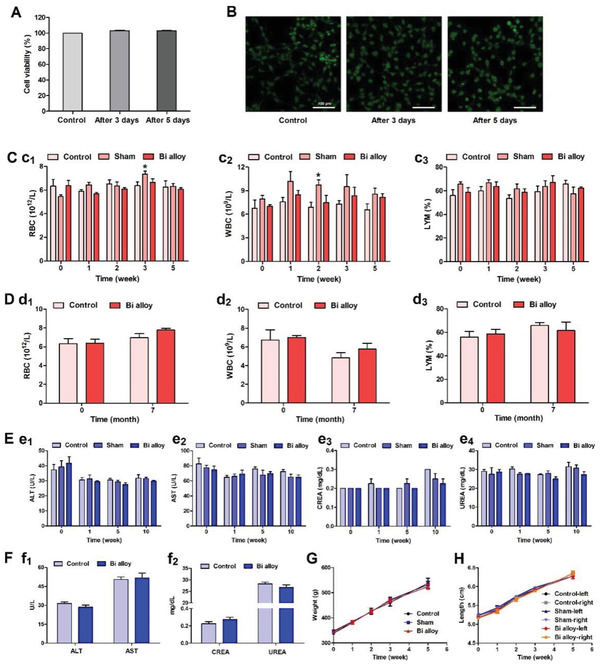
Toxicology evaluation of the Bi alloy in vitro and in vivo. A) The cell viability of BALB/c 3T3 cells incubated with the Bi alloy extract medium for 3 or 5 days (*n* = 7, *p* > 0.05). B) Live/dead cells image, scale bar 100 µm. C) The blood tests including (c_1_) red blood cells counts (RBC), (c_2_) white blood cells counts (WBC) and (c_3_) percentage of lymphocytes (LYM) after surgery with or without implanting Bi alloy to the left leg for 0, 1, 2, 3, 5 weeks (*n* = 5, *p < 0.05). D) The blood tests including (d_1_) RBC, (d_2_) WBC, and (d_3_) percentage of LYM after surgery with implanting Bi alloy to the left leg for 7 months (*n* = 5, *p* > 0.05). E) The biochemical tests about liver and kidney function including (e_1_) alanine aminotransferase (ALT), (e_2_) aspartate aminotransferase (AST), (e_3_) creatinine (CREA), and (e_4_) urea (UREA) levels in the blood after surgery with or without implanting Bi alloy to the left leg for 0, 1, 5, 10 weeks (*n* = 4, *p* > 0.05). F) The biochemical tests about liver function including (f_1_) ALT and AST and kidney function including (f_2_) CREA and UREA after implanting Bi alloy to the left leg for 7 months (*n* = 4, *p* > 0.05). G) Body weight (n = 5, *p* > 0.05) and H) leg length of rats after surgery with or without implanting Bi alloy to the left leg for 0, 1, 2, 3, 4, 5, 6 weeks (*n* = 5, *p* > 0.05). Results represent mean ± SEM in each group, *p*‐values are calculated using two‐tailed unpaired t‐test.

Biosafety is indispensable in the research and development of biomedical materials because such finally approved materials will be used for diagnosis and treatment of diseases. Therefore, the in vitro cytotoxicity of Bi alloy was tested first. The in vitro cytotoxicity of Bi alloy was detected by neutral red, live/dead cell staining and cell counting kit‐8 (CCK‐8). The Bi alloy was immersed in medium for 0, 3, and 5 days to prepare different extracts, and the BALB/c 3T3 fibroblasts (BALB/c 3T3 cells) was cultured in different extracts for 24 h. As shown in **Figure** **6**A, the neutral red staining indicating the cell viability for different extracts was ≈100%. In addition, the fluorescence image of the live/dead cells showed that the cell cultured in Bi alloy extracts for 3 or 5 days was alive and no obvious dead cells were observed (Figure 6B). To further test the direct cytotoxicity of Bi alloy in vitro, BALB/c 3T3 cells were incubated in cell medium with Bi alloy for 12, 24, and 48 h. The results of CCK‐8 detection showed that there were no significant differences compared with the control group (Figure S5A, Supporting Information). The cells around the Bi alloy grew well without obvious apoptosis under light microscope (Figure S5B, Supporting Information). Therefore, these results of in vitro cytotoxicity demonstrated that the Bi alloy is a low toxic biomaterial.

When implant materials are contacted with tissue, foreign body reaction might be induced, which triggers the aggregation of a large number of immune cells and inflammatory cytokines, inducing acute and chronic inflammatory response and fibrous reactions.^[^ ^36^ ^]^ Long‐term accumulation of negative factors will damage the function of liver and kidney.^[^ ^37^, ^38^ ^]^ Therefore, a series of experimental tests were needed to clarify the biocompatibility of Bi alloy in vivo. As shown in Figure 6C, routine blood test results showed that the red blood cell counts in the sham group were higher than the control group in the 3rd week (Figure 6Cc_1_), as well as, white blood cell counts in the sham group were higher than the control group in the 2nd week (Figure 6Cc_2_). The hemoglobin at the 3rd or 5th week, mean corpuscular volume at the 1st, 2nd, or 3rd week, and mean corpuscular hemoglobin at the 3rd week in the sham group were all higher than control group (Figure S6, Supporting Information). These results revealed that a large number of inflammatory factors were accumulated at the early stage of tissue injury, which basically returned to normal in the 5th week. Compared with the control group, no abnormalities were observed in blood routine indicators of Bi alloy group at the 1st, 2nd, 3rd, or 5th week (Figure 6C) and 7 months (Figure 6D and Figure S7, Supporting Information). This also revealed that the Bi alloy could remain in rats for 7 months without inflammation in blood. Therefore, the implantation of Bi alloy in the short or long‐term did not induce inflammatory reactions. In addition, liver and renal toxicity of blood biochemical criteria including alanine aminotransferase (ALT), aspartate aminotransferase (AST), creatinine (CREA), and urea (UREA) were performed in control, sham and Bi alloy group (4 rats in each group). The ALT, AST, CREA, and UREA levels among the groups showed no significant differences at 0, 1, 5, or 10 weeks (Figure 6E) and 7 months (Figure 6F), indicating that the long‐term implantation of Bi alloy did not cause hepatorenal toxicity in rats. It is worth noting that there was no effect on liver or kidney function of rats with Bi alloy implantation for 7 months. The growth status of the rats was normal (Figure 6G,H). This also demonstrated the excellent biocompatibility of Bi alloy.

## 3 Discussion

Millions of people around the world are encountering bone defects and acute or chronic pain caused by bone diseases such as trauma and bone tumors every year.^[^ ^6^ ^]^ High quality bone graft materials and effective relief of bone pain are the major challenges facing orthopedic science. In this study, a combined therapy with injectable Bi alloy (Bi_35_In_48.6_Sn_16_Zn_0.4_) was proposed to tackle bone defect repair and relieve pain under an AMF. The Bi alloy has a low melting point with 61.19 °C, which is much lower than that of traditional metals (more than 1000 °C). It achieves solid‐liquid phase transition smoothly, so as to facilitate the injection and solidification of Bi alloy used in bone defect repair. In comparison to rigid metal materials, the obtained Bi alloy material exhibited injectability and conformability. In addition, the Bi alloy owns excellent mechanical properties as a bone graft material, which is sufficient for filling and supporting bones. Besides, compared to other nonmetal materials, the Bi alloy can be observed in rats by Micro‐CT because of its radiopacity, which facilitates the observation and monitoring of Bi alloy after surgery. Although the implantation of bone graft materials is an effective method for bone defects repair, it is inevitable that risks such as, graft loosening, fracture, infection, and osteolysis may exist after implantation for a certain period of time.^[^ ^17^, ^18^, ^39^ ^]^ Hence, the necessary indicators including the integration of materials and bones, the inflammatory response of materials to surrounding tissues and the regeneration of bones with materials were tested to comprehensively evaluate the Bi alloy. The findings identified that Bi alloy remained in its original position after implantation for 210 days with Micro‐CT imaging, because it was firmly attached to bones. The integration ability of Bi alloy with bones was further proven by tissue sections. The specific staining of the sections showed that the Bi alloy had no significant inflammatory response to its surrounding bone tissues and did not affect the growth of the new tissue. Therefore, the Bi alloy is an excellent long‐term bone graft material with low trauma for bone defect repair.

In addition, the harmful stimulus from orthopaedic disease, such as, fracture osteomyelitis, bone marrow edema, osteoarthritis, and bone cancer induce hypersensitivity to pain.^[^ ^4^ ^]^ This is caused by stimulating the high expressing and activation of sensory receptors, such as, transient receptors on a large number of sensory neurons distributed around the periosteum or bone marrow. Opioids and non‐steroidal anti‐inflammatory drugs are commonly used as bone analgesics. However, the long‐term use of these drugs leads to respiratory suppression‐resistant nephrotoxic side effects easily and has adverse effects on bone remodeling/healing.^[^ ^4^, ^28^ ^]^ In order to reduce the side effects of drugs, thermal stimulation is being tried as an adjuvant therapy for the treatment of chronic back and shoulder pain, migraine, and primary pain.^[^ ^40^, ^41^, ^42^ ^]^ Gradually, thermal stimulation has evolved into the main analgesic therapy. For example, local thermal ablation or radiofrequency ablation with electrothermal needle is applied for tumor‐induced chronic or neuropathic pain, and acupuncture hyperthermia is used to treat pain and suppress inflammatory response. However, acupuncture hyperthermia leads to repeated traumatic tissue stimulation. Due to its excellent electrical and thermal conductivity, Bi alloy was applied to administrate remote (wireless) magnetic hyperthermia analgesia via AMF to reduce the damage to tissues. The effectiveness of this non‐contact magneto‐thermal analgesia method was demonstrated by mechanical and thermal pain sensitive behavioral experiments. In order to explore the mechanism of magnetic hyperthermia analgesia, immunohistochemical staining displayed that the magnetic hyperthermia of Bi alloy by AMF inhibits the expression of pain sensitization factors containing the IL‐6, SP, and TRPV1 and contributes to the relief of pain. Therefore, we speculate that the mechanism of magneto‐thermal analgesia might be through inhibiting the release of immune cytokines (IL‐1*β*, IL‐6) and the activity and expression of nociceptor receptors (TRPV1),^[^ ^43^ ^]^ eliminating the sensitization of primary nerve terminal nociceptor and reducing the release of neurotransmitter (Glutamate, SP),^[^ ^44^ ^]^ thereby reducing the transmission of pain signals to relieve pain. Furthermore, thermal stimulation of 40–44 °C has been reported to promote regeneration of bone tissue.^[^ ^45^, ^46^ ^]^ It is concluded that Bi alloy may accelerate the degree of bone healing in the process of wireless magneto‐thermal analgesia. Further elucidates the role of Bi alloy in bone defect repair and pain combined therapy. In addition, the Bi alloy has low immunogenicity compared to bioderived bone graft materials. Biocompatibility evaluation experiments demonstrated that the Bi alloy had low toxicity to normal cells in vitro and negligible toxicity liver and kidney in vivo. In particular, a vivo biocompatibility test showed that Bi alloy existed in the bone defect for up to 7 months without inducing hepatorenal toxicity, displaying its excellent biosecurity.

## 4 Conclusion

Overall, with low melting point, strong mechanical properties, excellent biocompatibility, and high quality bone affinity, Bi alloy achieves minimally invasive and lasting repair of bone defects to resolve the trauma caused by repeated revision. Meanwhile, the Bi alloy can be heated via remote contactless and wireless AMF due to its high electrical and thermal conductivity, which well tackles the problems of drug side effects for chronic pain induced by bone defects. This magnetic heat treatment method is feasible, comfortable, and noninvasive. Therefore, the Bi alloy is expected not only to repair bone defects, but also to relieve acute and chronic pains caused by bone defects with AMF exposure in the coming time.

## 5 Experimental Section

### Material and Preparation of Bi Alloy

The bismuth (Bi), indium (In), tin (Sn), and zinc (Zn) metals (purity of 99.99%) were prepared according to the mass ratio of 35%, 48.6%, 16%, and 0.4%, respectively. To prepare Bi alloy (Bi_35_In_48.6_Sn_16_Zn_0.4_), the weighed metals were put into crucibles in the vacuum with high‐temperature melting, and adjusted the power to 350–650 W through the control panel. The molten metal was stirred and shaken uniformly.

### Characterization of Bi Alloy

The melting point of Bi alloy was measured by differential scanning calorimetry (MicroCal PEAQ‐DSC, Malvin, America). The sample about 30 mg was placed in the Al_2_O_3_ crucible and put into the furnace of the DSC with the temperature changing rate of 10 °C min^−1^. The surface morphology of Bi alloy was obtained by SEM (SU‐8010, Japan), the distribution of metal elements was analyzed by EDS (Japan). As for mechanical strength, in order to measure the compressive hardness and elastic modulus of Bi alloy, four sites on the sample were pressed by the Nano indentation instrument (G200, Keysight Technology, China).

### Cytotoxicity Test

According to international standard ISO 10993‐12, the extract medium of Bi alloy was obtained by immersing the sample in complete medium (89% Dulbecco's modified Eagle's medium (DMEM), 10% fetal bovine serum, and 1% penicillin/streptomycin) with a ratio of 3 cm^2^ mL^−1^, then they were placed in a CO_2_ incubator at 37 °C for 3 and 5 days.^[^ ^22^ ^]^ To evaluate the biocompatibility of medical devices, the in vitro toxicity of medical devices can be detected by neutral red uptake according to the international standard ISO 10993–5.^[^ ^23^ ^]^ BALA/c 3T3 cells were suspended in untreated DMEM complete medium with the concentration of 1 × 10^5^ cells mL^−1^, then they were added into 96‐well plates and cultured in an incubator with 5% CO_2_ at 37 °C for 24 h. The medium was replaced with Bi alloy extract medium, and cells continued to be cultured for another 24 h. After that, the medium was replaced with 100µL NR medium for 3 h. They were washed once with 150 µL PBS, and 150 µL neutral red eluent were added. Finally, the collected sample was examined with a Spectra Max M5 (Molecular Devices, Inc., USA) and microplate assay (Varioskan Flash, Thermo, America) at an optical density (OD) value of 540 nm. In order to further evaluate the cytotoxicity of Bi alloy, live/dead cell staining was used to detect cell apoptosis. The suspension of BALA/c 3T3 with the concentration of 1 × 10^5^ cells mL^−1^ was cultured in 60 mm confocal dishes for 24 h. Then, the medium was replaced with Bi alloy extract medium. After 24 h, the medium was washed twice with PBS and followed by the addition of AO/EB (AO: EB = 1:1) solution. It was incubated avoidedly light for 2 min. Finally, the solution was replaced with PBS. The growth of cells was observed under the fluorescence microscope (Zeiss LSM710, China) by the excitation wavelength of 488 and 543 nm. Cell viabilities were determined using CCK‐8 assay. Cells were grown in absence of Bi alloy for 24 h. After that, cells were incubated with Bi alloy for 12 h, 24 h and 48 h, then the medium was removed and 100 µL of CCK‐8 medium solution was added to each well, in which cells were incubated for another 2 h. The absorbance at 450 nm was determined.

### Experimental Animals

Sprague‐Dawley (SD) rats (male, 8 weeks, Beijing Vital River Laboratory, China) were housed in a room maintained at daily light‐dark cycles at 25 °C. Rats were habituated to the testing paradigms for 3 days before data collection. The study protocols were approved by the Institutional Animal Care and Use Committee (IACUC) and followed by the Ethics Committee of Tsinghua University. All possible efforts were made to minimize unnecessary suffering of animals.

### Bone Defect Model and Bi Alloy Implantation

Rats were anesthetized by intraperitoneal injection of 2% pentobarbital (0.2 mL/100 g) according to their body weight. In the knee bottom, the bone tissue was exposed with a knife and cut blunt. And the bone defect model was constructed with a depth of about 10 mm via a 20 mL syringe needle. Then, the molten Bi alloy was implanted into the bone defect site. The muscles and skin were sutured when the Bi alloy was solidified. After the operation, the iodophor was daubed at the wound site. Analgesics and antibiotics were injected intraperitoneally to relieve pain and fight infection.

### In Vivo Toxicology Test

Blood routine and biochemical tests were used to detect the toxicology of Bi alloy in vivo. For blood routine measurement, the whole blood was collected from the tail vein of each SD rat after surgical operation for 0, 1, 2, 3, 5 weeks, and 7 months. And 500 µL whole blood of each SD rat was centrifuged (4 °C, 45 000 r min^−1^, 10 min) to obtain serum for liver and kidney function evaluation including UREA, CREA, ALT, and AST, after surgical operation for 0, 1, 5, 10 weeks, and 7 months.

### Histopathological Sections of Bone

The rat model bone was intercepted after the surgical operation for 8 months and the surrounding soft tissues were removed. Then they were fixed in 4% paraformaldehyde (PFA) for 1 week. Pour off the residual PFA and wash the bone with clean water. The bone was completely immersed in the mixed acid decalcified solution (the volume ratio of the bone to the decalcified solution was no less than 1:20) and fully permeated by magnetic stirring. The bone sample was placed in an automatic tissue dehydrator (Tissue‐TEK VIP) with gradient dehydration for 1–2 days after decalcification. The dehydrated bone sample was embedded by a paraffin embedding machine (Leica EG 1150H). The bone tissue slice was cut with a thickness of 5 µm by paraffin slicer (Leica RM 2245) and white slices were developed with a profiling machine (Leica HI 1210). The bonding of Bi alloy with bone tissues was observed by Nano Zoomer glass scan (Binson C10730‐12).

### Specific Staining of Bone Tissue Slices

The specific staining including hematoxylin‐eosin (HE), toluidine blue (TB), and Van Gieson (VG) was used to characterize the effect of Bi alloy on local bone tissue. The staining protocol was routine dewaxing of tissues into water, HE/TB/VG staining, gradient alcohol dehydration, xylene transparent, and neutral gum sealing.

### The Heating of Bi Alloy by Alternation Magnetic Field

Bi alloy heating was conducted with a magnetic induction heater (ZDBT‐2, Hanggong Electric Technology, Hangzhou). The input power was 1500 W and the output power was 750 W. The bone implanted with Bi alloy was placed 4.5 cm away from the square of the magnetic coil in vivo or in vitro. The magnetic induction heater was controlled with one cycle of 1 min containing 20 s pauses, and the heating process lasted for 20 min. The AMF heating procedure was monitored by a thermocouple.

### Paw Withdrawal Threshold Test

Rats were placed on the wire mesh and separated by plexiglass. The left posterior foot of rats was stimulated with different intensities of Von Frey cilium. The stimulation lasted for 5 s each time, and there were 5 times in succession with intervals of 30 s each time. The maximum stimulus intensity was 15 g. If there were 3 foot lifting on 5 stimulations, it is a positive result. And a lower grade of cilium was selected until finding the lowest cilium gram. The lowest Von Frey cilium gram was recorded as the PWT value.

### Paw Licking Latency Test

The PLL test was conducted by a hot plate measuring instrument (YLS‐21A, Ziyang Instrument, Tianjin). The constant temperature of the plate was kept at 55 °C. The time started when the rat was placed on this heating plate, and it stopped when the rat began licking its hind foot. This time was recorded as the PLL value. Each rat should not spend more than 35 s on the plate to avoid permanent tissue damage.

### Immunohistochemistry

5 days after bone defects modeling and AFM heating, rats were over‐anesthetized with 2% pentobarbital and perfused transcardially with saline, followed by 4% PFA. The left L4‐5 DRGs were dissected out, and post‐fixed in 4% PFA immediately for 24 h. The DRGs coated in paraffin were cut into 3 µm‐thick sections and performed the antigen retrieval of sections. Sections were incubated overnight in rabbit anti‐TRPV1 antibody (1:400, Bioss, China), rabbit anti‐IL‐6 antibody (1:500, Servicebio, China), or rabbit anti‐SP antibody (1:500, Servicebio, China) at 4 °C, which followed by incubation in secondary antibody (HRP labeled) from the corresponding species of primary antibody at room temperature for 50 min and reacted with diaminobenzidine (DAB) for coloration. In addition, the nucleus of sections was counterstained with hematoxylin stain solution for about 3 min. Immunohistochemical staining results were scanned with 3DHISTECH slice scanning system (Pannoramic SCAN, Hungary).

### Statistical Analysis

Data processing was carried out using GraphPad prism and Origin 8.6. All data in this paper was presented as mean ± SEM. Two‐tailed unpaired t‐test was employed to determine the level of significance with ns, * or #, ** or ##, ***, **** representative *p* > 0.05, *p* < 0.05, *p* < 0.01, *p* < 0.001, *p* < 0.0001, respectively.

## Conflict of Interest

The authors declare no conflict of interest.

## Supporting information

Supporting Information

## Data Availability

The data that support the findings of this study are available from the corresponding author upon reasonable request.
