# Supplementary material for: Injectable Affinity and Remote Magnetothermal Effects of Bi‐Based Alloy for Long‐Term Bone Defect Repair and Analgesia
Source: Adv Sci (Weinh). 2021 May 20;8(14):2100719. doi: 10.1002/advs.202100719 (PMC8292916; doi:10.1002/advs.202100719)
Supplement: Supplementary file 1 — Supporting Information [file ADVS-8-2100719-s001.pdf]

© 2021 Wiley-VCH GmbH

ADVANCED SCIENCE
Open Access

Supporting Information

for *Adv. Sci.*, DOI: 10.1002/advs.202100719

Injectable Affinity and Remote Magnetothermal Effects of Bi-basedAlloy for Long-term Bone Defect Repair and Analgesia

*Yuanyuan He, Yu Zhao, Linlin Fan, Xuelin Wang, Minghui Duan, Hongzhang Wang, Xiyu Zhu, Jing Liu**

Supporting information

Injectable Affinity and Remote Magnetothermal Effects of Bi-basedAlloy for Long-term Bone Defect Repair and Analgesia

*Yuanyuan He#, Yu Zhao#, Linlin Fan#, Xuelin Wang, Minghui Duan, Hongzhang Wang, Xiyu Zhu, Jing Liu**

Y. He, M. Duan, Dr. H. Wang, X. Zhu, Prof. J. Liu

Department of Biomedical Engineering, School of Medicine, Tsinghua University, Beijing 100084, China.

*E-mail: jliubme@tsinghua.edu.cn

Dr. L. Fan, Prof. J. Liu

Technical Institute of Physics and Chemistry, Chinese Academy of Sciences, Beijing 100190, China.

Dr. Y. Zhao

The Second Hospital of ShanXi Medical University, Shan Xi Medical University, Shan Xi030001,China.

Dr. X. Wang

School of Engineering Medicine, Beihang University, Beijing 100191, China.

Interdisciplinary Institute for Cancer Diagnosis and Treatment, Beijing Advanced Innovation Center for Biomedical Engineering, Beihang University, Beijing 100191, China.

#Y. He, Y. Zhao and L. Fan contributed equally to this work.

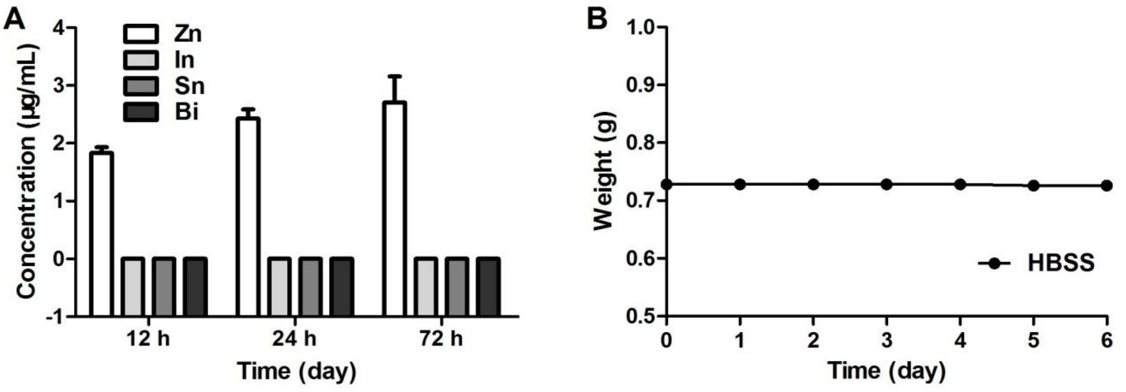

Figure S1. Stability of Bi alloy in HBSS solution. (A)Concentration of metallic elements released from Bi alloy in HBSS solution for 12 h, 24 h, and 72h (n=3). (B) Weight change of Bi alloy immersed in HBSS solution (n=5).Results represent mean ± SEM in each group, *P*-values are calculated using two-tailed unpaired t-test, $p > 0.05$.

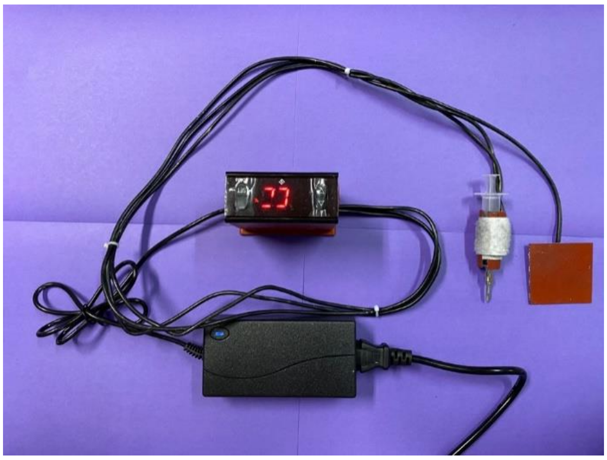

Figure S2. Heating device for syringe. The device consists of a 12/24V power adapter, a digital display temperature controller, a silica gel heating sleeve, a silica gel heating plate (red colors represents silica gel material) and a 5 ml syringe.

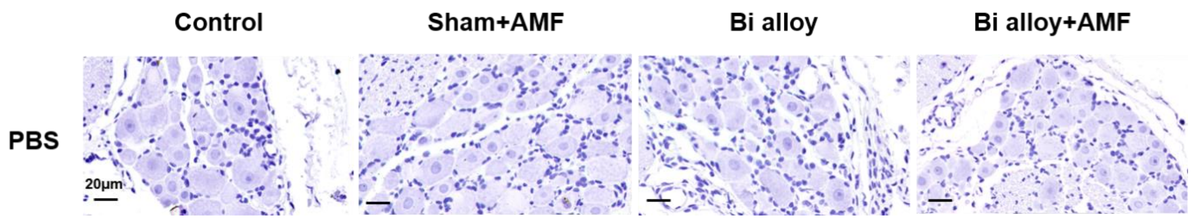

Figure S3. Negative (PBS staining)results of immunohistochemistryin L4-5 DRG after the treatment of pain by AMF or not among all groups.

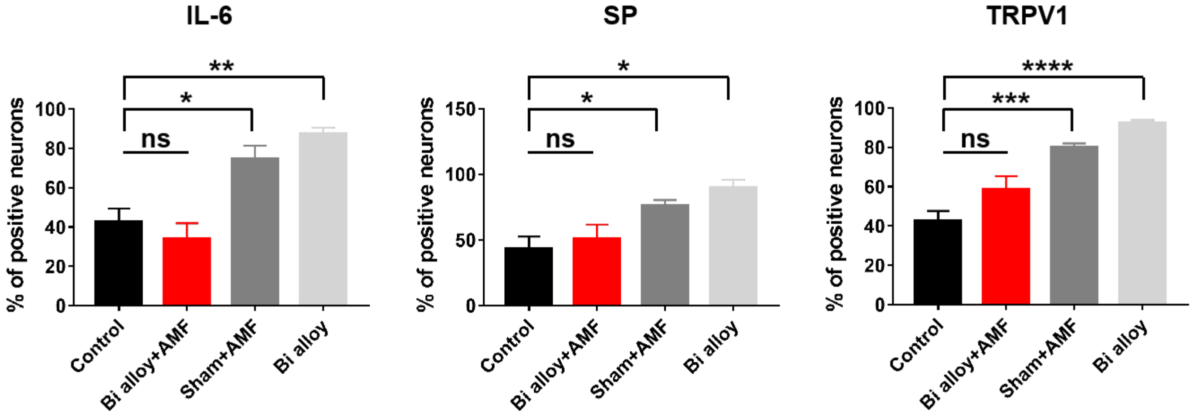

Figure S4. Quantification of expression levels of IL-6, SP and TRPV1by the percentage of positive neurons in L4-5 DRG after the treatment of pain by AMF or not among all groups. Results represent mean ± SEM in each group,the n value is the same as in the figure 5B,C,D, *P*-values are calculated using two-tailed unpaired t-test,ns $p > 0.05$, *$p < 0.05$, **$p < 0.01$, ***$p < 0.0005$, ****$p < 0.0001$, compared to naive control.

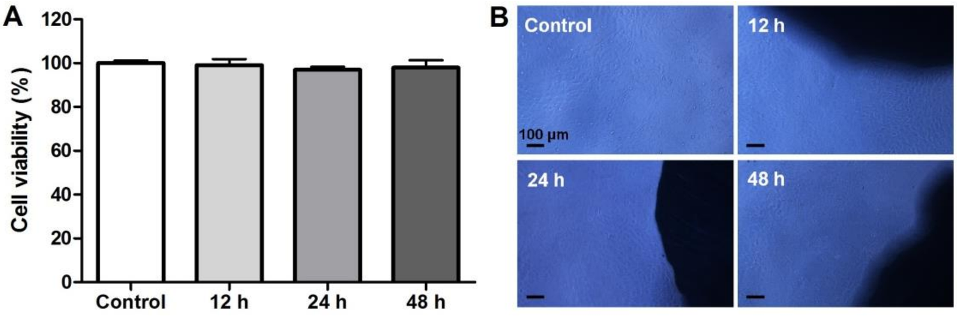

Figure S5. Direct cytotoxicity of the Bi alloy in vitro. (A) The cell viability of BALB/c 3T3 cells incubated with Bi alloy for 12 h, 24 h and 48 h.Results represent mean ± SEM in each group, n = 6, *P*-values are calculated using two-tailed unpaired t-test,$p > 0.05$.(B) The BALB/c 3T3 cell directly contacted and incubated with Bi alloy for 12 h, 24 h and 48 h.

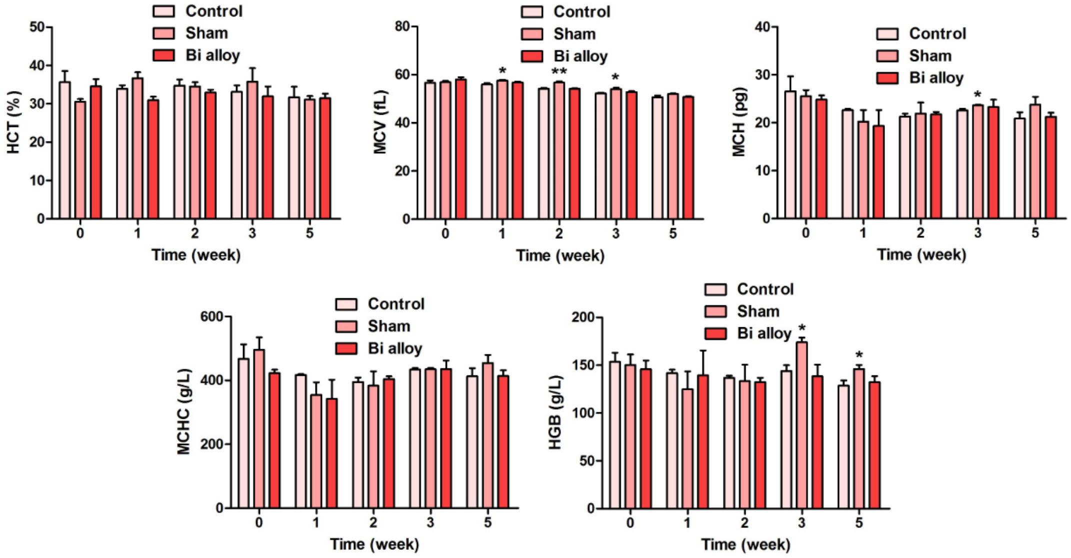

Figure S6. Short-term toxicology evaluation of the Bi alloy in vivo. The blood tests including hematokrit (HCT), mean corpuscular volume (MCV), mean corpuscular hemoglobin (MCH), mean corpuscular hemoglobin concentration (MCHC), hemoglobin (HGB) after surgery with or without implanting Bi alloy to the left leg for 0, 1, 2, 3, 5 weeks. Results represent mean ± SEM in each group, n = 5, *P*-values are calculated using two-tailed unpaired t-test, $*p < 0.05$, $**p < 0.01$.

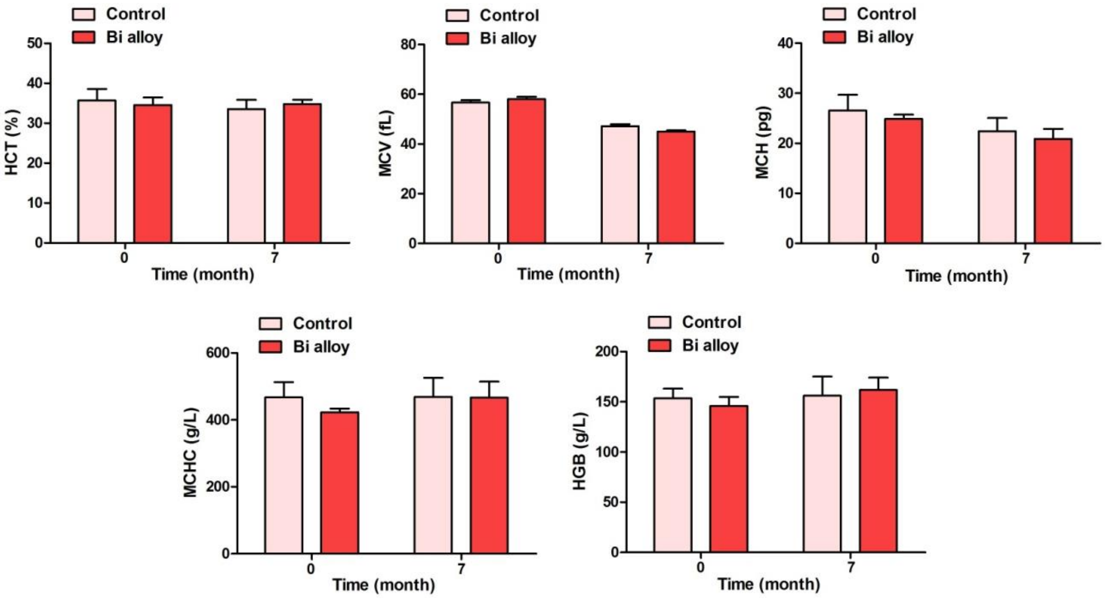

Figure S7. Long-term toxicology evaluation of the Bi alloy in vivo. The blood tests including hematokrit (HCT), mean corpuscular volume (MCV), mean corpuscular hemoglobin (MCH), mean corpuscular hemoglobin concentration (MCHC), hemoglobin (HGB) after surgery with implanting Bi alloy to the left leg for 7 months.Results represent mean ± SEM in each group, n = 5, *P*-values are calculated using two-tailed unpaired t-test,$p > 0.05$.
